# Nurse-like cells impact on disease progression in chronic lymphocytic leukemia

**DOI:** 10.1038/bcj.2015.108

**Published:** 2016-01-15

**Authors:** F Boissard, C Laurent, A G Ramsay, A Quillet-Mary, J-J Fournié, M Poupot, L Ysebaert

**Affiliations:** 1 CRCT, UMR1037, Inserm-Univ. Toulouse III Paul Sabatier-ERL5294 CNRS, Toulouse, France; 2 Department of Haematology, Institut Universitaire du Cancer de Toulouse - Oncopôle, Toulouse, France; 3 Department of Haemato-Oncology, Division of Cancer Studies, Faculty of Life Sciences & Medicine, King's College London, London, UK

Chronic lymphocytic leukemia (CLL) is one of the most common B-cell malignancies in adults, characterized by an accumulation of monoclonal CD5^+^ mature B cells in lymphoid tissues and peripheral blood.^1^ The complex interplay between malignant CLL cells and their surrounding bystander tumor microenvironment (TME)^2^ is critical for their survival and facilitates both their resistance to drug therapy and their orchestration of an immunosubversive milieu.^3^ CLL cells proliferate in distinct microanatomical tissue sites, termed proliferation centers, that allow intimate interactions with TME components notably nurse-like cells (NLC).^4^ These cells represent a leukemia-associated counterpart of tissue-associated macrophages, expressing high level of CD68 and CD163^5, 6^ and mediating chemoresistance particularly to ibrutinib, dexamethasone and chlorambucil.^7^ Even if these cells was well studied by *in vitro* approaches, the *in vivo* clinical impact of NLC outgrowth in the TME has not been definitively addressed to date in CLL, as described for the tumor-associated macrophages (TAMs) in solid tumors. If high TAM infiltration has been associated with a worse outcome in several solid cancers as well as in hematological malignancies including diffuse large B-cell lymphoma,^8^ in CLL studies were focused on indirect proof of evidence. Indeed, if CCL3 levels are associated with shorter treatment-free survival (TFS)^9^ and HMGB1 expression levels in the TME have been correlated with overall survival (OS),^10^ these prognostic factors do not clearly identify NLC infiltration as an important parameter associated with CLL outcome.

In the present study, we demonstrated that TME, particularly NLC, could impact CLL progression. For this purpose, we reported for the first time that (i) CD163^+^ NLC expression is correlated with CLL proliferation in lymph nodes (LNs), (ii) high soluble CD163 (sCD163) levels, the soluble counterpart of CD163, are linked with the worst prognostic factors in this disease, namely *TP53* mutations, complex karyotype and unmutated immunoglobulin heavy-chain variable (IGHV) status and (iii) high levels of sCD163 are associated with shorter TFS and OS.

To determine the clinical impact of NLC, we first compared the *in vivo* anatomical pattern of CD163^+^ macrophages in both healthy donor (reactive tonsils, Figure 1a) and CLL LNs tissue sections (Figure 1b). In normal LNs and tonsils, CD163^+^ macrophages were confined to the sub-capsular areas and along the lymphatic sinuses, whereas the B-cell zones were devoid of CD163^+^ cells, as previously reported. Moreover, in CLL LNs, CD163^+^ macrophages were present in the medulla, intertwined with leukemic cells and pseudofollicles. Confocal microscopy revealed that CD163^+^ cells were consistently CD68^+^ in CLL LNs, while some CD68^+^ macrophages were devoid of CD163 expression (Supplementary Figure 1). Because CD68 can also be expressed by non-myeloid cells,^11^ we hypothesized that CD68 staining might over-interpret the exact quantity of NLC in LNs and we decided to use CD163 as the most relevant marker for NLC.

We next questioned the reliability of CD163^+^ NLC within TME with regards to disease progression. We then performed immunohistochemistry analyses on 27 previously untreated CLL patients for whom nodal tissue biopsies were available in our institution. These patients had either a suspicion of disease transformation or were biopsied before CLL was evidenced from the immunophenotyping of circulating lymphocytes. As shown in Figures 1c and d, some patients (for example, patients 1 and 2) had few Ki-67^+^ proliferating CLL cells within pseudofollicles (and few CD163^+^ cells), and had indolent CLL. Notably, these watch and wait patients still had more CD163^+^ macrophages intertwined with tumoral B cells, as compared with normal LNs. In contrast, patients with active CLL (as defined by IWCLL2008 criteria) requiring therapy presented with (i) an increased frequency and size of pseudofollicles (for example, patients 3 and 4, Figures 1c and d), (ii) a higher fraction of Ki-67^+^ CLL cells and (iii) a significantly higher number of CD163^+^ cells. This is particularly relevant in the proliferating centers, as indolent CLL had very few CD163^+^ cells in these areas. Altogether, these data from a large cohort of 27 patients suggested that CD163^+^ cells load within the TME was significantly higher as CLL progressed towards first line of treatment.

In previous studies, sCD163 may be used as a reliable marker of the total pool of CD163^+^ cells in various conditions.^12^ Because of the limited availability of LN biopsies, and the absence of CD163 expression by circulating monocytes in CLL patients,^6^ we evaluated sCD163 levels (in serum) and other related soluble factor such as soluble CD68 (sCD68) and soluble HGMB1 (sHMGB1). Indeed, we found a higher amounts of sCD163, sCD68 and sHMGB1 in the sera of CLL patients than in age-matched normal donor sera (Figure 1e). Then, in this cohort of 94 patients naive to therapy (for thorough analysis of patient characteristics, see Supplementary Table 1), we correlated soluble factors levels with previously established prognostic markers. We found a statistically significant association between high sera levels of sCD163 and poor prognostic markers, such as unmutated IGHV status, complex karyotypes (defined by >3 anomalies), the presence of *TP53* mutations and a trend towards an association with del(17p) and *NOTCH1* mutational status (*P*=0.055; Table 1). This was very relevant as these markers are considered as the worst prognostic markers in this disease. On the other hand, we found no such correlation with Binet stage, suggesting aggressiveness of disease reflected more faithfully than ‘crude accumulation' of leukemic cells within tissues, and the amount of CD163^+^ macrophages in these patients (Supplementary Figure 2). We did not find any evidence of association between sCD68 or sHMGB1 levels and CLL prognostic factors (Table 1). Lymphocytosis (that is, circulating tumor bulk) or monocytosis (that is, putative precursors of NLCs once recruited to tumor niches) also had no impact on the levels of the three soluble factors studied (Supplementary Figure 2). Altogether, as sCD163 levels did not correlate with sCD68/HMGB1 levels (Supplementary Figure 2), our data suggest for the first time that aggressive CLL modulate their TME to increase the education of monocytes into CD163^+^ cells.

Of the 94 patients included in our study, 78 required treatment, according to the IWCLL2008 criteria for active disease, and 24 died during follow-up. Median time to first treatment in the entire cohort was short (36 months; Supplementary Tables 2 and 3). By receiver operating characteristic analysis we found that 1000 ng/ml of sCD163 was the best threshold to segregate patients into two groups. sCD163^high^ patients had significantly shorter TFS (logrank *P*=0.04, 30 versus 42 months; Figure 1f, Supplementary Table 2b), but also shorter OS (logrank *P*=0.03; Figure 1g, Supplementary Table 3). These last data are important, as most patients requiring therapy received modern immune-chemotherapy regimens (Supplementary Table 2b). On the other hand, sCD68 (high if >21 ng/ml) and sHMGB1 (high if >227 ng/ml) were not associated with outcomes (Supplementary Figures 2 and 3). Interestingly, this increase of sCD163 levels in patient sera can be found in other hemopathy including Hodgkin lymphoma,^13^ a previous publication (though exploring a smaller CLL cohort) showed very similar levels as ours, both in CLL patients or healthy donor (suggesting we are not studying a too-selected panel of samples).^14^ In the former study, using a higher threshold (2.45 mg/l) to predict only TFS (not OS), the authors pointed towards a role of CD163 without functional data with NLC, as we present in this manuscript.^14^ Furthermore in our cohort, high levels of sCD163 are linked to well-established adverse biological factors, such as IGHV mutational status as previously described,^14^ but also the presence of *TP53* mutations and complex karyotype, suggesting that leukemic cells with high-risk features raise more CD163^+^ cells within their TME, an as yet unanswered question in the field of TAMs in oncology. In univariate analyses, sCD163 levels predicted TFS with SF3B1mutated status, normal fluorescence *in situ* hybridization and Binet stage (Supplementary Table 2). In univariate analyses for OS, sCD163, deletion of chromosome 17p and *TP53* mutated status were predictive (Supplementary Table 3). Altogether, our data suggest that, being associated with *TP53* mutations, IGHV unmutated status and complex karyotypes, sCD163 is TME-related risk factor associated with shorter survival after frontline immunochemotherapy, and therefore should be investigated in independent cohorts of patients. Even if the size of the cohort precludes from drawing too strong statements in terms of OS particularly, these deaths were all CLL related. sCD163 has been indeed related to survival in a wide variety of medical conditions, outside the cancer setting. Moreover, despite we confirmed the increased sHMGB1 levels published by Jia *et al.*^10^ in CLL sera, in our cohort sHMGB1 or sCD68 levels cannot be linked to prognostic markers or outcome. These results reinforce the prominence of CD163 or its soluble counterpart to determine in future studies the burden and clinical impact of NLC in disease course in a large cohort of LNs or sera, as previously published in myeloma.^15^

In summary, we have demonstrated for the first time that CD163 and its soluble counterpart sCD163 are the best markers for future studying of NLC *in vivo* and *in vitro*. If future studies are needed to definitely identify CD163^+^ NLC (or sCD163) as the first TME-related prognostic factor having clinical impact in CLL, targeting therapies against these cells are promising approaches to overcome TME pro-survival capacities in CLL.

## Figures and Tables

**Figure 1:**
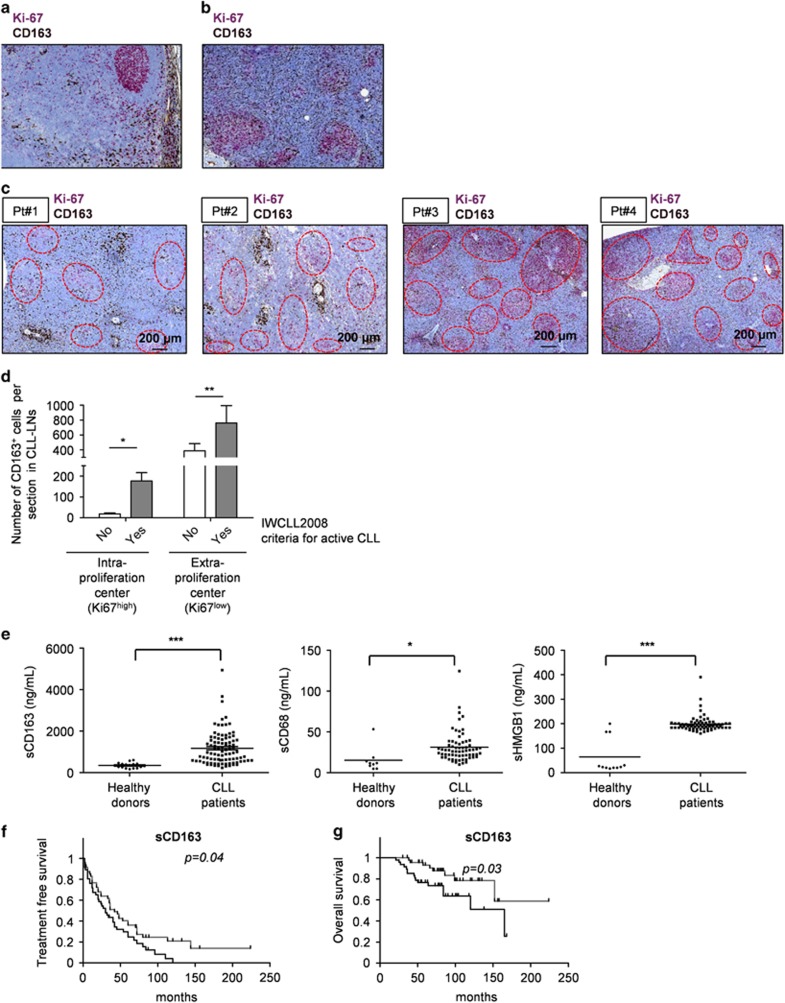
High soluble CD163 levels correlate with shorter treatment-free survival (TFS) and shorter overall survival (OS). (**a**) CD163^+^ staining from one representative (*n*=7) healthy donor lymph node tissue section. CD163^+^ cells were present in the lymphatic sinuses and along the sub-capsular area, but were excluded from the germinal center remnants (pink) in the normal lymph node (× 200). (**b**) CD163^+^ staining from one representative (*n*=27) CLL lymph node tissue section. CD163^+^ cells (brown) were spread throughout the parenchyma, infiltrating proliferation centers (pink) in the CLL lymph node (× 200). CD163^+^ cells were stained with a CD163 antibody (brown) and germinal center cells/proliferating CLL cells with a Ki-67 antibody (pink). (**c**) Representative pictures showing the density and distribution of CD163^+^ in CLL LN from two patients with indolent CLL (Pt #1 and #2), and two patients with therapy-urging CLL (Pt#3 and #4; × 100). (**d**) According to disease aggressiveness, the number of CD163^+^ cells both outside and within proliferating centers significantly differs, suggesting that the number of NLCs within the TME may be used for prognostication purposes. NLCs were stained with a CD163 antibody (brown) and proliferation centers with a Ki-67 antibody (pink). Relative quantification of Ki-67^+^ cells or CD163^+^ cells was made by two-blinded separate analyses. (**e**) Quantification (by enzyme-linked immunosorbent assay testing) of soluble CD163 (left), soluble CD68 (center) or soluble HMGB1 (right) in serum from healthy donors (•) or CLL patients (□) shows a significant increase of all three markers only in CLL patients (mirroring NLCs–CLL interactions in the TME). Soluble HMGB1 levels show very few disparities among patient samples, whereas s.d.'s of the CD163 and CD68 dosages are broader. (**f**, **g**) Kaplan–Meier curves showing the probability of (**f**) TFS and (**g**) OS. CLL patients were divided into groups of patients with low (<1000 ng/ml, gray curve) or high (>1000 ng/ml, black curve) levels of sCD163. These categories were determined by receiver operating characteristic curves. *P<0.05, **P<0.01, ***P<0.001.

**Table 1:** Correlation between levels of sCD163, sCD68 or sHMGB1 and established prognostic markers in CLL

*Characteristics*	*Category*	*Median sCD163 (range)*	P*-value* *(CD163)*	*Median sCD68 (range)*	P*-value* *(CD68)*	*Median sHMGB1 (range)*	P*-value* *(HMGB1)*
Age	<65 years	887.4 (326.4–2312)		26.54 (10.1–26.5)		192.3 (167.3–390.7)	
	>65 years	985 (302–4944)	0.6619	28.14 (11.6–124.6)	0.1918	190.7 (160.7–274.0)	0.482
Sex	Female	686 (375.2–3427)		28.30 (15.9–73.5)		190.7 (160.7–274.0)	
	Male	1098 (235–4944)	0.2586	26.16 (10.1–124.6)	0.3106	190.7 (167.3–390.7)	0.4563
IGHV status	Mutated	669 (302–4944)		22.3 (11.6–124.6)		190.7 (160.7–274)	
	Unmutated	1308 (235-2662)	0.0031	28.1 (12.7–73.5)	0.3858	194 (167.3–390.7)	0.5336
Cytogenetics	Tri 12	1227 (376–1558)	0.1152	25.2 (12.7–28.4)	0.8279	180.7 (174.0–197.3)	0.2823
	Del(13q)	946 (375.2–2359)	0.7053	30.4 (15.9–68.9)	0.1632	190.7 (174.0–214.0)	0.9749
	Del(11q)	597 (565–4944)	0.6617	31.1 (15.9–52.9)	0.5963	200.7 (174–390.7)	0.4194
	Del(17p)	1775 (995-1922)	0.0743	17.5 (15.5–19.4)	0.2142	187.3 (184.0–190.7)	0.4897
	Complex karyotype	184 5 (1162–1922)	0.0236	18.4 (15.5–19.4)	0.0799	189.0 (184.0–190.7)	0.1658
Recurrent mutations	*SF3B1*	1308 (621.1–2298)	0.2611	32.3 (18.2–68.9)	0.5839	185.7 (167.3–204.0)	0.3607
	*NOTCH1*	1562 (416–4944)	0.0555	32.8 (12.7–52.9)	0.1399	197.3 (184.0–254.0)	0.0718
	*TP53*	1764 (995–4944)	0.0093	35.1 (15.5–80.0)	0.4309	190.7 (180.7–204.0)	0.9766

Abbreviations: Del, deletion; IGHV, immunoglobulin heavy-chain variable segment mutational status; tri 12, trisomy 12.

In our series, high sCD163 levels were found to be correlated with *TP53* mutational status, unmutated IGHV and complex karyotypes, the three most predictive variables for survival (progression-free survival and OS) in CLL. A trend towards significance was also seen with del(17p) and *NOTCH1* mutations, suggesting that these CLL cases modulate their TME towards an increase in CD163^+^ cells.
