# Supplementary material for: Nurse-like cells impact on disease progression in chronic lymphocytic leukemia
Source: Blood Cancer J. 2016 Jan 15;6(1):e381–. doi: 10.1038/bcj.2015.108 (PMC4742625; doi:10.1038/bcj.2015.108)
Supplement: Supplementary Information [file bcj2015108x1.doc]

**Supplemental method**

**Immunohistochemistry and confocal microscopy**

Samples from CLL lymph node biopsies or reactive tonsils fixed in 10% buffered formalin were retrieved from the tissue bank of the department of pathology at the Purpan Hospital, Toulouse, between 2000 and 2011. The use of these samples (n=27), was validated by the institutional review board of the Toulouse University Hospital. Specimens were fixed and embedded in paraffin then processed for routine histopathological examination.

For immunohistochemistry, 3 µm-thick sections were stained with hematoxylin and eosin, CD163 primary antibody (clone 10D6, dilution 1:100; DAKO) and Ki67 primary antibody (clone MIB-1, dilution1:100 Dako). Sections were then scanned using brightfield scan mode with a 20X/NA0.80 Zeiss Plan-Apochromat dry objective and images were acquired with a two megapixel 3CCD color camera (CIS Cam RefVCC-F52U25CL, CIS Americas INc., Tokyo, Japan), achieving a 0.22 μm/pixel resolution, corresponding to a x56.09 magnification at the highest optical resolution in conventional microscopy. After having manually delineated the proliferation center areas in CLL lymph nodes or germinal centers in reactive lymph nodes using Ki67 staining on each digitalized slide, CD163 staining of each LN sample was manually scored by an expert pathologist.

For confocal microscopy, samples were pre-treated by microwave incubation in pH 6.0, 0.1 M sodium citrate and then permeabilized with 0.1% saponin (in PBS containing 3% BSA/HEPES, 10% goat serum), and incubated overnight at 4°C with the following two primary antibodies: CD68 (clone PGM1; dilution 1:20; DAKO) and CD163 (clone 10D6, dilution 1:10; DAKO) in PBS containing 3% BSA/HEPES, 0.1% saponin. Primary antibodies were followed by goat anti-mouse isotype-specific antibodies labelled with Alexa 488 and Alexa 555 (Molecular Probes) for 2 hours at RT. Samples were mounted in Fluorescence Mounting Medium® (DAKO) and examined using a Zeiss LSM 710 confocal microscope (Zeiss, Germany) with a 63x Plan-Apochromat objective (1.4 oil)*.* An argon laser at 488 nm was used to detect the Alexa 488 fluorochrome. To detect Alexa 555 fluorescence a helium laser was filtered at 543 nm. Under standard imaging conditions no signal from one fluorochrome could be detected with the other filter set. For each pair of antibodies used, standardized conditions for pinhole size, and for gain and offset (brightness and contrast), were used for image capture.

**Serum collection and ELISA**

Sera used were obtained from patients during their first visit to our center. Peripheral blood samples were drawn using a BD Vacutainer (BD Biosciences), and serum was collected after centrifugation and stored at -80°C before use.

ELISA from soluble CD163 (sCD163) (RD systems, UK), soluble HMGB1 (sHMGB1) (Cusabio, Wuhan, Hubei Province, China) and soluble CD68 (sCD68) (Wuhan EIAab Science, Wuhan, China) were realized on patient sera or on age-matched healthy donor sera, according to the manufacturer’s recommendations.

**Determination of IGHV status, FISH abnormalities and recurrent mutations**

Fluorescence in situ hybridization (FISH) and IGHV mutational analysis, were realized at the University Hospital of Purpan,

The mutation hotspots of the *TP53* (exons 4-9; RefSeq NM_000546.5), *SF3B1* (exons 14, 15, 16; RefSeq NM_012433.2) and *NOTCH1* (exon 34; RefSeq NM_017617.2) genes were screened by polymerase chain reaction (PCR), followed by high resolution fusion in 10 µL on a LightCycler LC 480 (Roche, Basel, Switzerland) (details of primers are provided in Supplemental Table 1). For *TP53* and *SF3B1*, recurrent mutations were validated by Sanger sequencing (3130 XL Genetic Analyzer, Applied Biosystems, Foster City, CA) of positive amplicons. For patients with a 17p deletion, *TP53* Sanger sequencing was systematically performed. The recurrent mutation delCT of *NOTCH1* (c.7544_7545delCT) was validated by capillary electrophoresis of amplicons. *TP53* mutations and/or *TP53* deletion were pooled together as *TP53* alterations.

**Statistical analysis**

Continuous variables were summarized with descriptive statistics, such as mean, SD, median, and range. Categorical variables were tabulated with frequency and percentage. The Fisher’s exact test was used to evaluate the association between categorical variables; the Wilcoxon rank-sum test was applied to compare each of the continuous variables between two different categories of dichotomized factors. Spearman correlation coefficients were computed to evaluate the correlation between two continuous variables.

The treatment free survival (TFS) was defined as the time from diagnosis to the date of first therapy if patients had received any, or was otherwise censored on the last visit date. The overall survival (OS) was defined as the time from diagnosis to the date of death if patients died or was otherwise censored on the last visit. TFS and OS were estimated by the Kaplan and Meier method and assessed by the log-rank test using Statistica ® software.

Throughout this process, clinical and laboratory variables were retained only if their Wald P values were <0.05. (* p<0.05, ** p<0.01, *** p<0.001). The statistical analyses were performed with PRISM software.

**Supplemental Figure 1**

**Supplemental Figure 1: Co-expression of CD163 and CD68 in CLL lymph nodes.** Tissue sections of CLL lymph nodes were stained for CD68 (green) and CD163 (red) then viewed by confocal microscopy revealed that CD68^+^ cells are CD163^+^ or CD163^-^, suggesting staining of myeloid cells other than true NLC, and therefore prompting the use of CD163 staining to track NLC *in vivo* (Bar = 50µm). One representative experiment (of 3) is shown.

**Supplemental Figure 2**


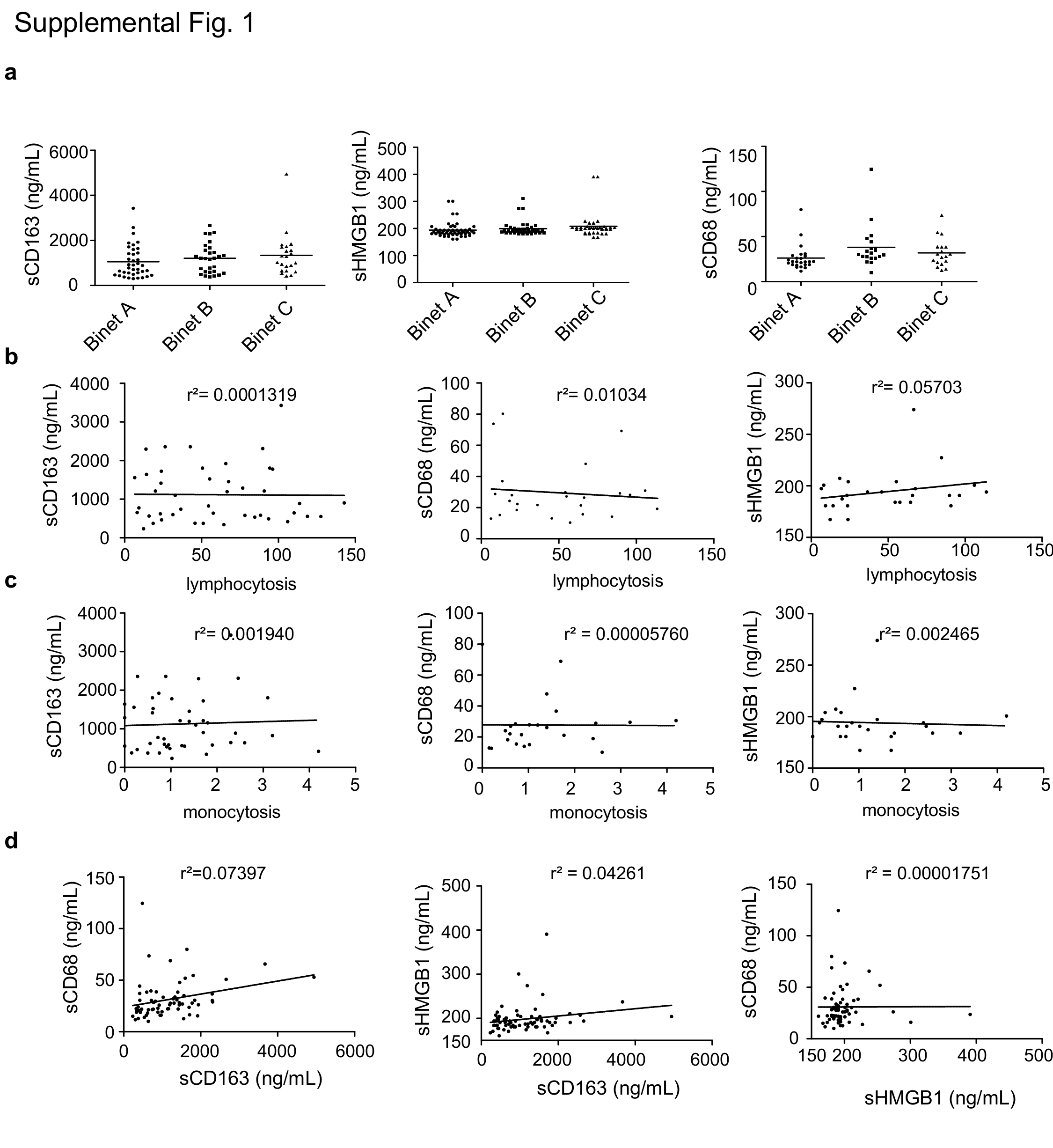


**Supplemental Figure 2: No relationship exists between sCD163 (left), sCD68 (center), sHMBG1 (right) and Binet Stage (A), lymphocytosis (B), or monocytosis (C). No relationship was found between each of the soluble markers (D).**

**Supplemental Figure 3**

**Supplemental Figure 3: No relationship exists between sCD68 (left) or sHMGB1 (right) and clinical outcome**. Kaplan-Meier curves showing the probability of treatment-free survival **(A)**, and overall survival **(B)**. CLL patients were divided into groups of patients with low (blue curves) or high (red curves) levels of sCD68 or sHMGB1 (high levels taken as >21ng/mL or >227ng/mL, respectively). These categories were determined by ROC curves.

**Supplemental Table 1**

| **Characteristics** | **Category** | **n (%)** |
| --- | --- | --- |
| **Sex** | **Female** | 29 (30.9) |
|  | **Male** | 65 (69.1) |
| **Binet Stage** | **A** | 38 (40.4) |
|  | **B** | 29 (30.9) |
|  | **C** | 22 (23.4) |
| **IGHV status** | **Mutated** | 44 (46.8) |
|  | **Unmutated** | 38 (40.4) |
| **Cytogenetics** | **Tri 12** | 13 (13.9) |
|  | **Del(13q)** | 29 (30.9) |
|  | **Del(11q)** | 11 (11.7) |
|  | **Del(17p)** | 6 (6.4) |
|  | **Complex karyotype** | 15 (15.9) |
| **Recurrent mutations** | ***SF3B1*** | 5 (5.3) |
|  | ***NOTCH1*** | 14(14.9) |
|  | ***TP53*** | 8 (8.5) |

**Supplemental table 1: Clinical characteristics of 94 patients with CLL.** Del: deletion, IGHV: immunoglobulin heavy chain variable segment mutational status, tri 12: trisomy 12.

**Supplemental Table 2**

**A**

| Caracteristics | Category | Total | Patients treated | Median TTFT, mo | p |
| --- | --- | --- | --- | --- | --- |
|  | All patients | 94 | 78 | 36 | - |
| **Binet Stage** | A | 38 | 27 | 54 | - |
|  | B | 29 | 26 | 30 | **<0.001** |
|  | C | 22 | 22 | 22.5 | **<0.001** |
| **IgHV status** | Mutated | 38 | 31 | 43.5 | - |
|  | Unmutated | 44 | 38 | 25 | 0.12 |
| **Cytogenetics** | Normal FISH | 12 | 9 | 76 | **0.05** |
|  | Tri 12 | 13 | 13 | 30 | 0.27 |
|  | Del(13q) | 29 | 26 | 32 | 0.42 |
|  | Del(11q) | 11 | 10 | 20 | 0.65 |
|  | Del(17p) | 6 | 3 | 60 | 0.28 |
|  | Complex karyotype | 15 | 14 | 27 | 0.54 |
| **Recurrents mutations** | *SF3B1* | 5 | 5 | 13 | **0.05** |
|  | *NOTCH1* | 14 | 13 | 38 | 0.63 |
|  | *TP53* | 8 | 6 | 24 | 0.16 |
| **sCD163** | Low (<1000 ng/ml) | 47 | 37 | 42 | - |
|  | High (>1000 ng/mL) | 48 | 41 | 30 | **0.04** |

B

| **Received treatment** | **Patients treated (%)** |
| --- | --- |
| All | 78 (100%) |
| Rituximab + fludarabine + cyclophosphamide | 40 (51.29) |
| Rituximab + fludarabine | 11 (14.11) |
| Rituximab + cyclophosphamide + dexamethasone | 8 (10.27) |
| Rituximab + cyclophosphamide + pentostatine | 5 (6.41) |
| Rituximab + bendamustine | 3 (3.84) |
| Gemcitabine + alentuzumab | 3 (3.84) |
| Fludarabine + cyclophosphamide + alentuzumab | 2 (2.56) |
| Rituximab + chlorambucil | 1 (1.28) |
| Rituximab + mini CHOP (cyclophosphamide + hydroxyadriamycine + vincristine + predinsone) | 1 (1.28) |
| Rituximab mini CHV (cyclophosmamide + hydroxyadriamcycine + etoposide) | 1 (1.28) |
| Chlorambucil | 1 (1.28) |
| Fludarabine + cyclophosphamide | 1 (1.28) |
| Mini CHOP | 1 (1.28) |

**Supplemental Table 2: Treatment Free Survival (TFS) according to the different prognostic subgroups (A) and breakdown of drugs received at the first treatment (B).** In our series, only B/C Binet stage, the presence of the *SF3B1* mutation or high sCD163 correlated with shorter TFS. Del: deletion, IgHV: immunoglobulin heavy chain variable segment mutational status, tri 12: trisomy 12.

**Supplemental Table 3**

| **Caracteristics** | **Category** | **Total** | **Patients deceased** | **p** |
| --- | --- | --- | --- | --- |
|  | All patients | 94 | 24 | - |
| **Binet Stage** | A | 38 | 7 | - |
|  | B | 29 | 5 | 0.34 |
|  | C | 22 | 6 | 0.59 |
| **IgHV status** | Mutated | 38 | 9 |  |
|  | Unmutated | 44 | 6 | 0.42 |
| **Cytogenetics** | Normal FISH | 12 | 5 | 0.26 |
|  | Tri 12 | 13 | 3 | 0.76 |
|  | Del(13q) | 29 | 9 | 0.46 |
|  | Del(11q) | 11 | 2 | 0.77 |
|  | Del(17p) | 6 | 3 | **0.05** |
|  | Complex karyotype | 15 | 4 | 0.85 |
| **Recurrents mutations** | *SF3B1* | 5 | 1 | 0.97 |
|  | *NOTCH1* | 14 | 3 | 0.80 |
|  | *TP53* | 8 | 5 | **<0.01** |
| **sCD16**3 | Low (<1000 ng/mL) | 47 | 8 | **-** |
|  | High (>1000 ng/mL) | 48 | 16 | **0.03** |

**Supplemental Table 3: Overall survival (OS) according to the different prognostic subgroups.** In our series, only the presence of the deletion 17p, the *TP53* mutation and high sCD163 correlated with shorter OS. Del: deletion, IgHV: immunoglobulin heavy chain variable segment mutational status, tri 12: trisomy 12.
